# Exosome-Mediated Enhancement of Fat Graft Retention: A Comparative Preclinical Study with Stromal Vascular Fraction

**DOI:** 10.1007/s00266-025-05350-5

**Published:** 2025-11-17

**Authors:** Ying Zhu, Ki Yong Hong, Hak Chang

**Affiliations:** 1 Department of Plastic and Reconstructive Surgery, Seoul National University College of Medicine, Seoul, Korea; 2 Department of Plastic and Reconstructive Surgery, Seoul National University Hospital, Seoul National University College of Medicine, 101 Daehak-ro, Jongno-gu, Seoul, 03080 Korea

**Keywords:** Fat graft, Adipose-derived stromal cells, Stromal vascular fraction, Exosome, Regenerative medicine

## Abstract

**Background:**

Cell-assisted lipotransfer (CAL) is a fat grafting technique that enhances graft survival by supplementing grafts with autologous stromal vascular fraction (SVF) or adipose-derived stromal cells (ASCs). However, its clinical translation has been hindered owing to inherent biological variability, potential cell viability issues, tumorigenic risk, and the complex regulatory landscape associated with cell-based therapies. To overcome these challenges, exosome has gained increasing attention as a promising non-cellular therapeutic modality capable of preserving key regenerative functions while minimizing relative concerns. This study aimed to compare the efficacy of SVF-enriched lipotransfer with fat co-transplanted with

exosomes from ASCs to identify a reliable and clinically applicable non-cellular strategy for optimizing fat graft outcomes.

**Methods:**

In vivo, minced human fat tissue mixed with phosphate-buffered saline, SVF cells, or exosomes was grafted into nude mice. Grafts were evaluated through microcomputed tomography at weeks 4 and 8 post-grafting and histological analysis at weeks 1 and 8 post-grafting.

**Results:**

The exosome group showed a significantly higher volume retention rate than the control group at weeks 4 and 8 post-grafting. Histological analysis revealed that exosomes exhibited more pronounced effects in reducing inflammation, preserving perilipin-positive adipocytes, and promoting angiogenesis in the grafted fats compared with those of SVF cells.

**Conclusions:**

Our findings highlight the potential of exosomes in improving fat graft retention and overall tissue regeneration, suggesting that fat co-transplanted with exosomes from ASCs could serve as a superior alternative to SVF-enriched lipotransfer.

**No Level Assigned:**

This journal requires that authors assign a level of evidence to each submission to which Evidence-Based Medicine rankings are applicable. This excludes Review Articles, Book Reviews, and manuscripts that concern Basic Science, Animal Studies, Cadaver Studies, and Experimental Studies. For a full description of these Evidence-Based Medicine ratings, please refer to the Table of Contents or the online Instructions to Authors www.springer.com/00266

**Supplementary Information:**

The online version contains supplementary material available at 10.1007/s00266-025-05350-5.

## Introduction

Cell-assisted lipotransfer (CAL) is an innovative technique where autologous fat grafting is integrated with cell therapies to overcome critical limitations of conventional fat grafting, such as high resorption rates and inconsistent long-term outcomes [1]. The multipotent stromal vascular fraction (SVF) cells or adipose-derived stromal cells (ASCs) introduced during CAL can differentiate into adipocytes and endothelial cells and directly integrate into the graft tissue [2, 3]. Additionally, these cells secrete a wide range of growth factors, immunomodulatory agents, and cytokines that modulate the microenvironment, promoting angiogenesis and improving fat graft survival during tissue remodeling [4–6].

Stem cells create a supportive microenvironment for fat graft survival; however, there are still concerns regarding their tumorigenic potential [7, 8]. Replicative stress increases the propensity for genomic instability and abnormal differentiation [9], which combined with their self-renewal properties, increases the risk of uncontrolled proliferation and potential tumor formation. Additionally, intercellular competition can cause severe inflammation, impairing angiogenesis and contributing to fat graft degeneration [10]. Furthermore, the stringent regulatory environment significantly limits the use of stem cell therapies, underscoring the need for a more controllable non-cellular alternative to CAL [11].

Exosomes from ASCs are essential paracrine components that carry various bioactive molecules, including proteins, lipids, and non-coding RNAs. The vital cellular functions of ASCs, such as promoting cell proliferation, migration, immunoregulation, and angiogenesis, are primarily mediated by exosomes [12, 13]. However, the focus of current research has largely been on exosomes as stem cell derivatives, without fully recognizing their potential as independent therapeutic agents [12]. This oversight has resulted in a lack of comprehensive comparative studies across various biological materials, thereby preventing a complete understanding of exosomes and limiting their translation into effective clinical applications.

Owing to the safety concerns and regulatory restrictions associated with the clinical application of expanded ASCs, SVF has become a more accessible and minimally manipulated alternative for tissue repair [14–16]. Given its adipose origin and its established application in cell-assisted therapies, it provides a reliable clinical benchmark for assessing the regenerative potential of exosomes from ASCs. Therefore, the aim of this study was to compare the efficacy of SVF-enriched lipotransfer with fat co-transplanted with exosomes from proliferating ASCs in improving fat graft outcomes, thereby exploring a promising non-cellular alternative to CAL.

## Materials and Methods

### Preparation and Characterization of Exosomes

This study was conducted in accordance with the principles of the Declaration of Helsinki and was approved by the institutional review board and institutional animal care and use committee of the authors’ institution. Abdominal fat was collected from discarded tissues from three healthy female patients who had undergone autologous breast reconstruction. To obtain ASCs, SVF cells and ASCs were isolated and sub-cultured using a previously described method [17]. A working cell bank was established with ASCs from passages 1–6.

To harvest exosomes from ASCs, when ASCs reached 70–80% confluence, the culture medium was removed, and the cells were washed with phosphate-buffered saline (PBS) to minimize interference from exosomes derived from fetal bovine serum. Subsequently, the medium was replaced with Dulbecco’s Modified Eagle Medium (Thermo Fisher Scientific, Waltham, MA) supplemented with 1% penicillin-streptomycin (Capricorn Scientific, Ebsdorfergrund, Germany). After 48 h, the cell culture supernatant was collected and initially centrifuged at 2,000 × *g* for 20 min in an ultracentrifuge (Optima XE-1000; Beckman Coulter, Brea, CA) to remove cellular debris. Thereafter, the supernatant was centrifuged at 10,000×*g* for 30 min to obtain the clarified supernatant. After filtration through 0.22-μm filters, the supernatant underwent two additional rounds of ultracentrifugation at 120,000×*g* for 70 min. The temperature was maintained at 4 °C throughout the centrifugation process. The pellet was resuspended in PBS, quantified using a bicinchoninic acid protein assay, and stored at − 80 °C.

The size distribution and nanoparticle density were assessed using a Nanosight NS300 (Malvern Panalytical, Malvern, United Kingdom). Exosome morphology was observed using a transmission electron microscope (TEM) (JEM-1400; JEOL Ltd., Tokyo, Japan). Western blot analysis was performed using CD63, CD81, TSG101 (Abcam, Cambridge, United Kingdom), and α-tubulin (Santa Cruz Biotechnology, Dallas, TX).

### Cell Internalization Assay

To confirm the capacity of exosomes to internalize into ASCs *in vitro*, exosomes were labeled with PKH26 (Sigma-Aldrich, St. Louis, MO) and co-incubated with ASCs for 48 h. Following fixation with 4% paraformaldehyde for 30 min, cells were stained with Phalloidin-FITC Reagent (Abcam) and DAPI (Thermo Fisher Scientific). Stained samples were subsequently observed, and images were captured using a fluorescence microscope (DMI4000B; Leica Microsystems, Wetzlar, Germany).

### Cell Viability Assay

To determine an effective exosome concentration for *in vivo* experiments, an *in vitro* cell viability assay was performed using various exosome concentrations. In total, 6 × 10^3^ ASCs in complete culture medium were seeded into each well of a 96-well plate. After overnight incubation, the medium was replaced and supplemented with exosomes at concentrations of 100 or 200 μg/ml for the experimental groups, whereas no exosome supplementation was conducted for the control group. Cell viability was assessed at 0, 24, 48, and 72 h using a Cell Counting Kit-8 (CCK-8) assay (Dojindo, Kumamoto, Japan). The optical density (OD) value at 450 nm was measured with a microplate reader (Molecular Devices, San Jose, CA), and the cell viability rate was calculated using the following formula:

$$Cell \,viability \,\left(\% \,of \,control\right) = \left[\left(Ae - Ab\right) \div \left(Ac - Ab\right)\right] \times 100\%$$

where Ae, Ab, and Ac represent the OD values of the experimental well, blank well, and control well.

### Fat Grafting

Athymic nude mice (male, 7 weeks old, 20–25 g) were purchased and acclimatized for 7 days in a well-ventilated and temperature-controlled environment (24 ± 2 °C) with a 12-h light/dark cycle before the commencement of the experiment. To determine the optimal SVF-cell concentration for SVF-enriched lipotransfer, we initially investigated the enrichment effect of different SVF-cell numbers on fat graft retention. Minced adipose tissue (200 µl) mixed with 1 × 10^3^, 1 × 10^4^, 1 × 10^5^, and 1 × 10^6^ SVF cells was injected subcutaneously into the dorsal area of three randomly selected 8-week-old mice. Micro-computed tomography (micro-CT) scans were acquired immediately after fat grafting and at 8-week post-grafting. The graft volume was assessed using 3-matic Research 13.0 software (Materialise NV, Leuven, Belgium). The graft volume retention rate at 8 weeks was calculated using the following formula:

$$8-week\, volume\, retention\, rate\, of \,fat \,graft = {(V}_{8th} \div { V}_{0}) \times 100\%$$

where V_8th_ represents the volume measured at 8 weeks after fat grafting and V_0_ represents the fat volume measured immediately after fat grafting.

Next, based on the optimal exosome concentration obtained from the cell viability assay and optimal SVF-cell concentration determined from fat grafting experiments using various SVF-cell concentrations cells, 15 mice were randomly divided into control, SVF, and exosome groups. Subsequently, minced adipose tissue (200 µl) mixed with PBS, SVF cells, and exosomes was injected into the supraperiosteal plane of the mice using an 18-gauge needle. CT was performed immediately after the fat grafting procedure and at 4- and 8-week post-grafting. Five mice from each group were sacrificed at 8-week post-grafting, and the grafted fats were harvested and processed for further analysis.

To track the biodistribution and cellular uptake of exosomes *in vivo*, PKH26-labeled exosomes were co-transplanted with fat into nude mice. Fluorescence signals were monitored weekly using the IVIS Spectrum imaging system (PerkinElmer, Waltham, MA) for up to 8-week post-grafting. At week 1, graft tissues were collected and subjected to whole-mount immunofluorescence (IF) staining [3] with perilipin (Invitrogen, Carlsbad, CA), CD31 (Abcam), F4/80 (Abcam), and DAPI (Thermo Fisher Scientific).

### Histological Analysis

Following a previously established protocol, hematoxylin and eosin (H&E) staining of paraffin-embedded tissue sections was performed to quantify the percentage area of normal adipocytes, fibrosis, inflammation, and oil cysts [18]. For IF staining, tissue sections were incubated with the following primary antibodies diluted in blocking solution overnight at 4 °C: perilipin (Invitrogen), CD31 (Abcam), F4/80 (Abcam), INOS (Invitrogen), and CD206 (R&D Systems, Minneapolis, MN). After incubating with secondary antibodies, the nuclei were stained with DAPI (Thermo Fisher Scientific). Images of H&E staining results were captured using a light microscope (ECLIPSE Ci-L; Nikon, Tokyo, Japan), while IF staining results were obtained using a confocal microscope (TCS SP8; Leica, Wetzlar, Germany). All measurements were performed in five high-power fields (100× or 200× magnification) using ImageJ software (National Institutes of Health, Bethesda, MD).

### Statistical Analysis

Each experiment was conducted at least in triplicate. For comparison between the two groups in the CCK-8 assay, an independent samples *t*-test was performed. For data that were not normally distributed and had a small sample size, data are expressed as the median and interquartile range (IQR). The nonparametric Kruskal–Wallis test, followed by Bonferroni-corrected post hoc Mann–Whitney U tests, was used for statistical analysis. All analyses were conducted using SPSS version 26.0 (IBM Corp., Armonk, NY). A *p*-value of < 0.05 was considered statistically significant.

## Results

### Characterization of Exosomes

**Fig. 1:**
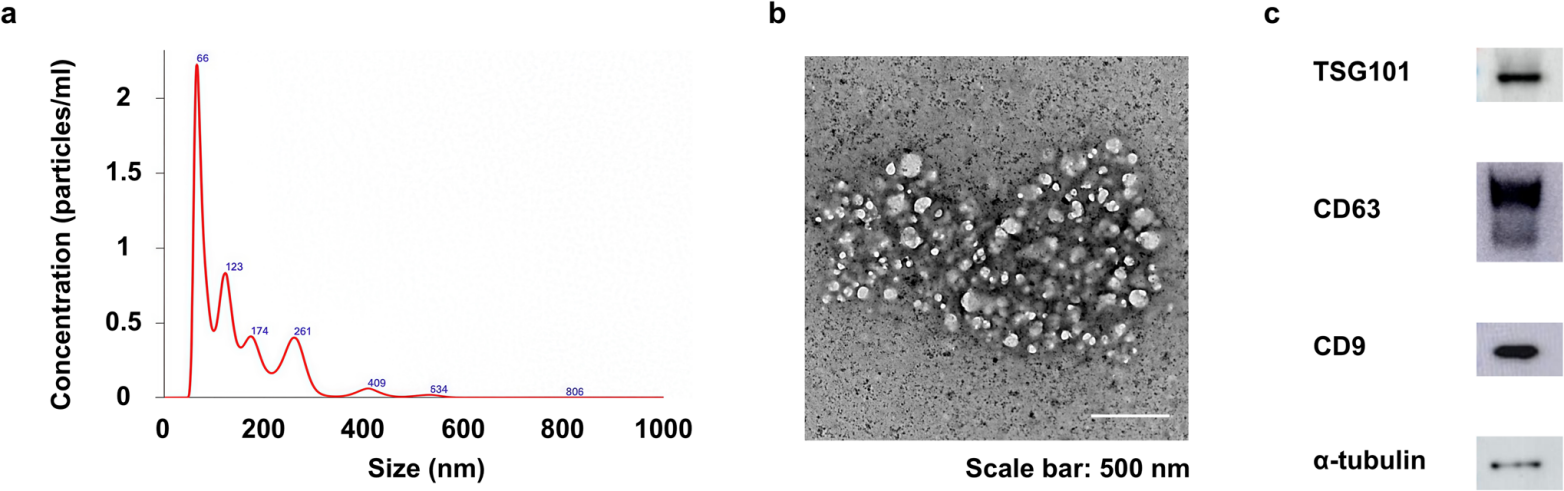
Characterization of exosomes. **a** Nanoparticle tracking analysis was used to estimate the size distribution of exosomes. **b** Transmission electron microscope photomicrographs of exosomes (scale bar = 200 nm). **c** Western blot analysis of TSG101, CD63, CD9, and α-tubulin expression in exosomes.

The pellet obtained after ultracentrifugation appeared as a semitransparent gelatinous precipitate with a light brownish-yellow color. The exosome production rate was approximately 0.5 µg/ml of the conditioned medium from ASC cultures. Nanoparticle tracking analysis showed a mean exosome diameter of 148.1 ± 92.2 nm, with a peak distribution at 65.6 nm (Fig. 1a). In the TEM images, the exosomes were depicted as spherical membranous vesicles with a phospholipid bilayer structure, smaller than 200 nm in diameter (Fig. 1b). Western blot analysis revealed pronounced expression of CD9, CD63, and TSG101 in exosomes (Fig. 1c). These findings confirmed the successful isolation of exosomes from ASCs.

### Improved Adipose-Derived Stromal Cell Viability by Exosome Uptake

**Fig. 2:**
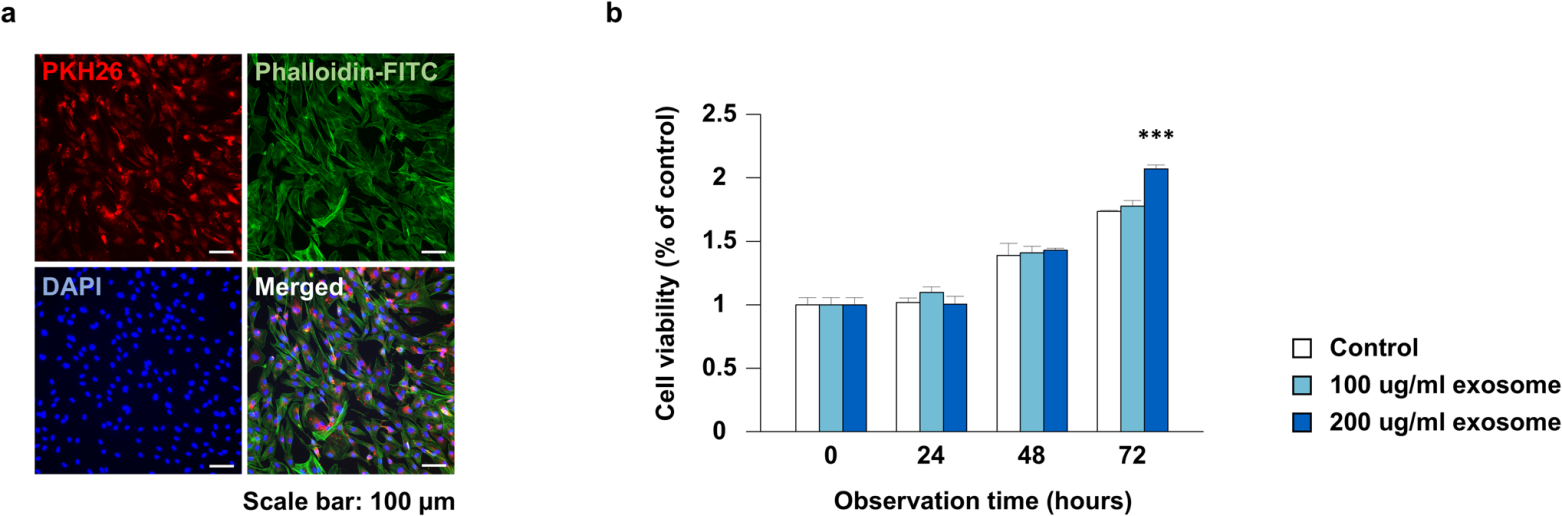
Cellular internalization of exosomes and their effect on cell viability over time. **a** Immunofluorescent images of adipose-derived stromal cells (ASCs) co-incubated with PKH26-labeled exosomes for 48 h. The cytoskeleton of ASCs is labeled with Phalloidin-FITC, and the nuclei are labeled with DAPI. Exosomes are visible in the perinuclear region of ASCs (scale bar = 100 μm). **b** The effect of different concentrations of exosomes on the cell viability of adipose-derived stromal cells is quantified with optical density values using the Cell Counting Kit 8 assay. At 72 h after treatment, the cell viability rates in the 200-µg/ml exosome groups were significantly increased compared with those in the control group (*p* < 0.001). ***p < 0.001 versus control group.

After 48 h of co-incubation with ASCs and PKH26-labeled exosomes, we observed concentrated PKH26-red fluorescence around the cell nuclei, suggesting successful exosome delivery into cells (Fig. 2a). Exosome internalization increased ASC viability, with a gradual increase in cell viability rate in the experimental group compared with that of the control group at 48 h. At 72 h, cell viability in the exosome group at 200 µg/ml was significantly improved compared with that in the control group (*p* < 0.001) (Fig. 2b), reaching twice the baseline level. Based on the greater improvement observed, the concentration of 200 µg/ml was selected for further investigation.

### Quantitative Analysis of Grafted Fats

The effect of different SVF-cell concentrations on fat graft retention was evaluated, revealing the highest retention at an optimal concentration of 1 × 10^5^ SVF cells (see Supplemental Figure 1). This optimal concentration was subsequently used for the following experiment.

**Fig. 3:**
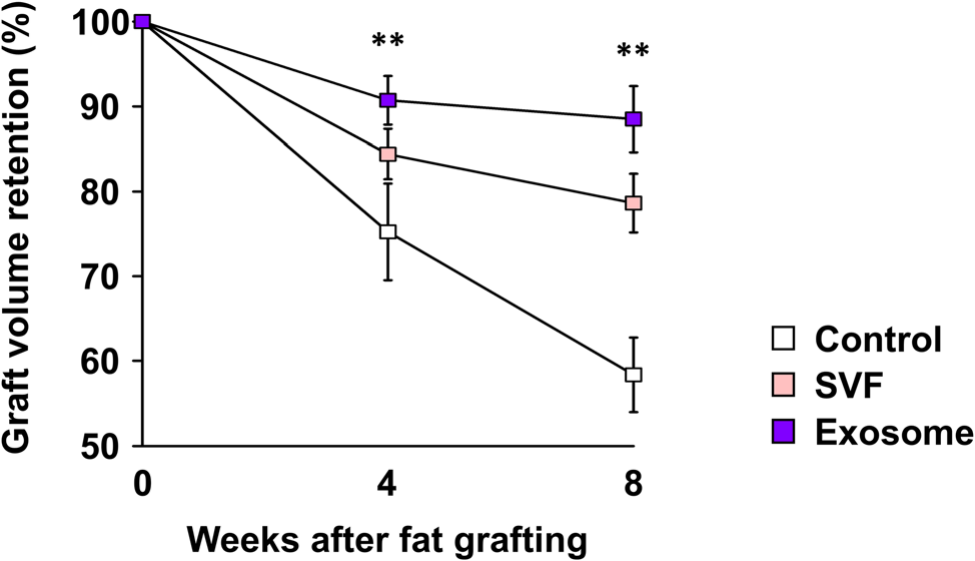
Changes in the volume retention rate of grafted fats over 8 weeks. A significantly higher volume retention rate is observed in the exosome group than in the control group (*p =* 0.007 at 4 weeks and* p* = 0.002 at 8 weeks). **p < 0.01 versus the control group. SVF, stromal vascular fraction.

At 4- and 8-week post-transplantation, fat graft retention in the exosome group [4 weeks, median, 91.3% (IQR, 88.1–93.0%); 8 weeks, median, 90.3% (IQR, 85.3–90.8%)] was significantly higher than that in the control group [4 weeks, median, 73.5% (IQR, 70.6–80.7%), *p* = 0.007; 8 weeks, median, 59.5% (IQR, 53.5–62.6%), *p* = 0.002]. While the SVF group [4 weeks, median, 84.6% (IQR, 81.6–87.1%); 8 weeks, median, 78.2% (IQR, 75.2–82.3%)] also showed higher retention than that of the control; however, the difference did not reach statistical significance at either time point (Fig. 3).

### Exosomes Improve the Quality of Grafted Fats More Effectively than Stromal Vascular Fraction Cells

**Fig. 4:**
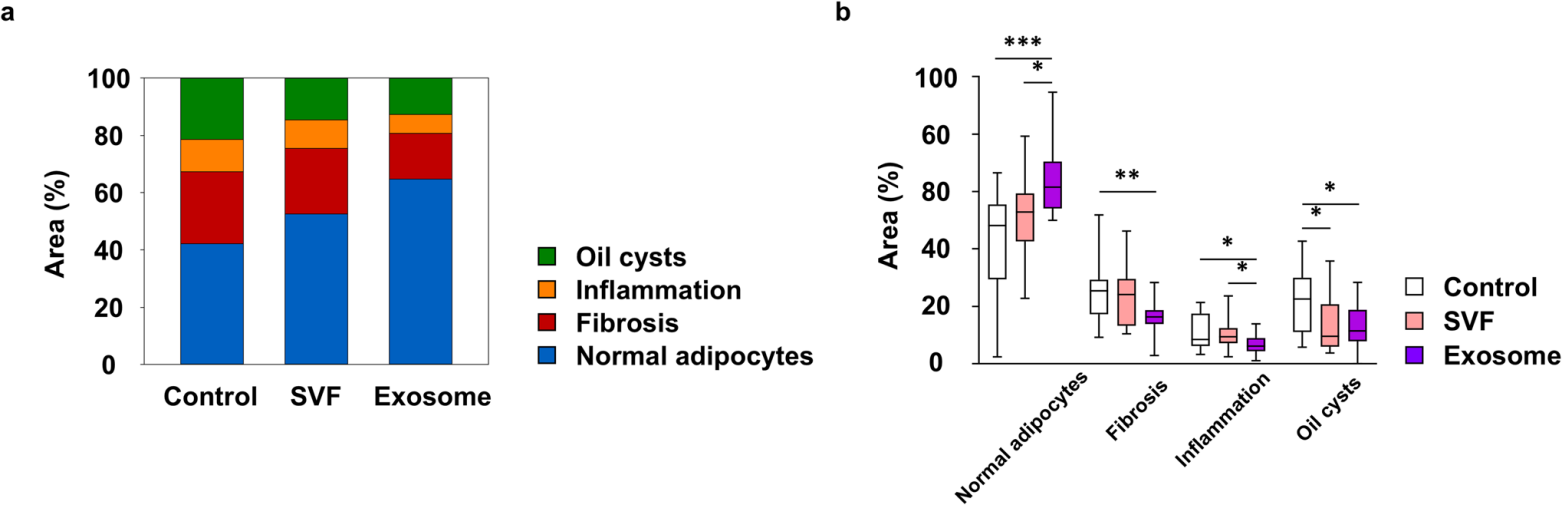
Histological analysis of fat grafts at 8 weeks post-grafting using hematoxylin and eosin staining. **a** Percentage area of normal adipocytes, fibrosis, inflammation, and oil cysts; **b** Quantitative evaluation of percentage area of normal adipocytes, fibrosis, inflammation, and oil cysts. *p < 0.05, **p < 0.01, ***p < 0.001. SVF, stromal vascular fraction.

Qualitative analyses were conducted using H&E slides to evaluate the proportions of fibrosis, inflammation, oil cysts, and adipocytes (Fig. 4a). Compared with that of the control group, the exosome group showed a significant reduction in fibrosis [control group, median, 25.4% (IQR, 17.3–29.3%); exosome group, median, 16.3% (IQR, 13.9–18.7%); *p* = 0.004], inflammatory infiltration [control group, median, 8.4% (IQR, 6.2–17.5%); exosome group, median, 6.2% (IQR, 4.4–9.0%); *p* = 0.014] and large oil cysts formation [control group, median, 22.6% (IQR, 11.1–30.0%); exosome group, median, 11.5% (IQR, 7.8–18.8%); *p* = 0.031], as well as a significantly higher proportion of normal adipocytes [control group, median, 48.1% (IQR, 29.5–55.5%); exosome group, median, 61.5% (IQR, 54.1–70.4%); *p* < 0.001]. When compared with that of the SVF group, the exosome group exhibited no significant difference in fibrosis [SVF group, median, 24.2% (IQR, 13.3–29.6%)] and cyst formation [SVF group, median, 9.6% (IQR, 5.9–20.7%)] but showed significantly greater adipocyte preservation [SVF group, median, 52.9% (IQR, 42.5–59.3%); *p* = 0.014] and a comparable reduction in inflammatory infiltration [SVF group, median, 9.4% (IQR, 7.2–12.5%); *p* = 0.024]. Notably, compared with that of the control group, the SVF group exhibited significant improvement only in reducing cyst formation (*p* = 0.041), with no substantial differences observed in adipocyte preservation, fibrosis, or inflammatory infiltration (Fig. 4b). These findings indicate that exosome exerts more pronounced effects on enhancing adipocyte retention, mitigating inflammation and fibrosis, and minimizing cyst formation, highlighting their superior potential over SVF in improving fat graft quality (see Supplemental Figure 2).

### Exosomes Enhance Fat Graft Survival and Vascularization More Effectively than Stromal Vascular Fraction Cells

Visual inspection showed significant differences in macroscopic appearance among the three groups, with the exosome group exhibiting a more extensive vascular network on the graft surfaces compared with those of the other two groups (see Supplemental Figure 3). Notably, severe inflammation was observed in one case in the control group, whereas a paired case in the exosome group, receiving a graft from the same donor under identical processing conditions, showed improved structural integrity and a visibly higher graft survival rate (see Supplemental Figure 4).

**Fig. 5:**
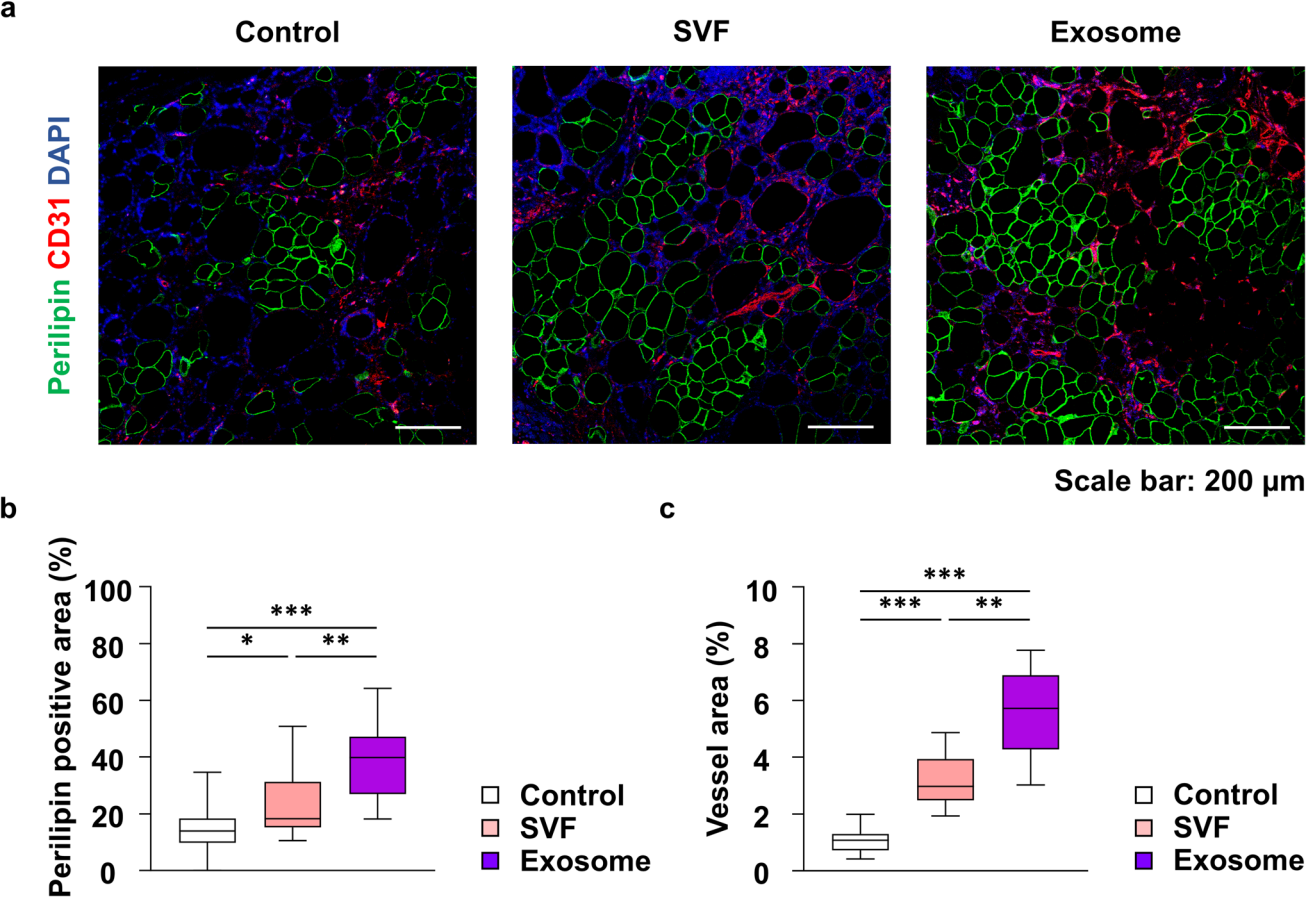
Immunofluorescent staining was conducted to evaluate adipocyte preservation and angiogenesis in fat grafts at 8 weeks post-grafting. **a** Viable adipocytes and blood vessels in graft tissues were identified using immunofluorescent staining for perilipin (green) and CD31 (red) (scale bar = 200 μm). **b** Quantification of perilipin-positive adipocyte area in the grafted tissues. **c** Quantification of vessel area in the grafted tissues. *p < 0.05, **p < 0.01, ***p < 0.001. SVF, stromal vascular fraction.

To further validate these histological findings, IF staining was conducted to evaluate adipocyte preservation and angiogenesis via perilipin and CD31 staining (Fig. 5a). The percentage of the perilipin-positive area was significantly higher in the exosome group [median, 39.8% (IQR, 26.8–47.2%)] than in both the control [median, 13.9% (IQR, 9.8–18.4%); *p < 0.001*] and SVF [median, 18.3% (IQR, 15.2–31.2%); *p* = 0.025] groups. Regarding neovascularization, the percentage of CD31-positive area was significantly increased in the exosome group [median, 5.7% (IQR, 4.3–6.9%), *p* < 0.001] and SVF group [median, 3.0% (IQR, 2.5–3.9%), *p* = 0.003] compared with that in the control group [median, 1.1% (IQR, 0.7–1.3%)]. Furthermore, the exosome group exhibited a significantly greater vascular density than the SVF group (*p* < 0.001) (Fig. 5b, c).

### Exosomes Exhibit Superior Immunomodulatory Effects by Promoting M2 Polarization and Reducing M1 Infiltration

**Fig. 6:**
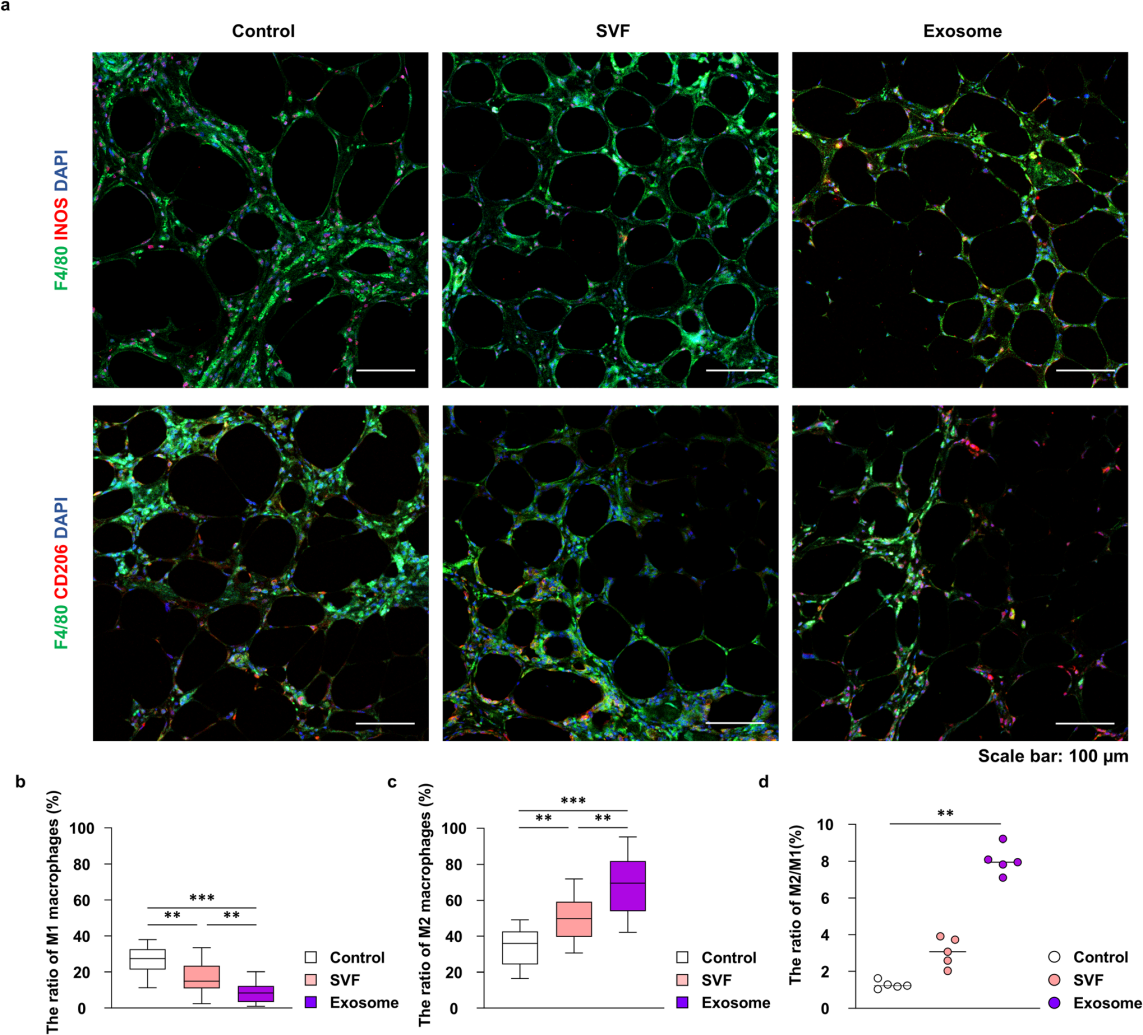
Immunofluorescent staining of M1 and M2 macrophages in fat grafts at 8-week post-grafting. **a** M1 macrophages in grafted tissues were confirmed using immunofluorescence staining of F4/80 (green) and inducible nitric oxide synthase (INOS) (red). M2 macrophages in grafted tissues were confirmed using immunofluorescence staining of F4/80 (green) and CD206 (red) (scale bar = 100 μm). **b** Quantification of M1 macrophage proportion within grafted tissues. The ratio of M1 macrophage proportion was calculated as the percentage of F4/80^+^ INOS^+^ cells among total F4/80^+^ macrophages. **c** Quantification of M2 macrophage proportion within grafted tissues. The ratio of M2 macrophage proportion was calculated as the percentage of F4/80^+^ CD206^+^ cells among total F4/80^+^ macrophages. **d** The ratio of M2 to M1 macrophages. **p < 0.01, ***p < 0.001. SVF, stromal vascular fraction.

To evaluate the immunomodulatory effects of different treatments, macrophage polarization was further analyzed using IF staining, with M1 macrophages defined as F4/80^+^ iNOS^+^ cells and M2 macrophages as F4/80^+^ CD206^+^ cells (Fig. 6a). The proportion of M1 macrophages among F4/80^+^ cells was significantly reduced in the SVF [median, 15.0 (IQR, 10.9–23.4); *p* = 0.002] and exosome [median, 8.4 (IQR, 3.4–12.3);* p* < 0.001] groups compared with that in the control group [median, 27.4 (IQR, 21.3–32.6)] and was also significantly lower in the exosome group than in the SVF group (*p* = 0.007) (Fig. 6b). For M2 macrophages, the exosome group [median, 69.7 (IQR, 53.9-81.9)] showed a significantly higher percentage than did the control [median, 35.9 (IQR, 24.2–42.6); *p* < 0.001] and SVF [median, 49.8 (IQR, 39.6–59.2); *p* = 0.002] groups (Fig. 6c). Furthermore, the M2/M1 ratio was significantly elevated in the exosome group [median, 8.0 (IQR, 7.5–8.7)] compared with that in the control group [median, 1.2 (IQR, 1.1–1.5); *p* = 0.001], whereas no significant difference was observed between the SVF group [median, 3.1 (IQR, 2.3–3.8] and other groups (Fig. 6d). These findings reveal that exosome treatment more effectively promotes M2 polarization and suppresses M1 infiltration than SVF cells, supporting its superior immunomodulatory capacity in fat grafting.

### Uptake of PKH26-labeled Exosomes In Vivo

Based on the longitudinal fluorescence imaging, exosomes persisted stably in the graft site for at least 4 weeks, with the fluorescence signal gradually fading and disappearing entirely by week 8 (see Supplemental Figure 5).

**Fig. 7:**
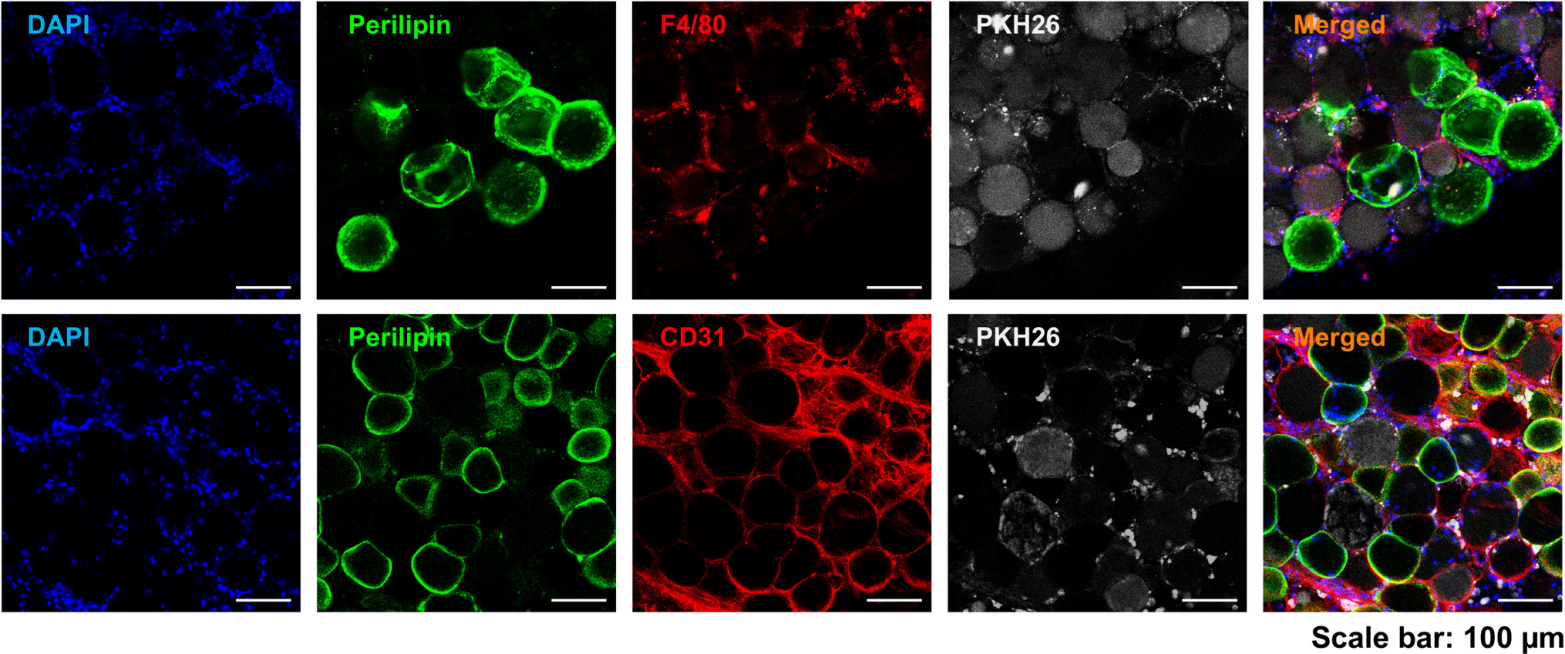
At 1-week post-grafting, the PKH26-labeled exosomes were detected within the cells in grafted tissues. Viable adipocytes were identified through perilipin staining, macrophages were labeled with F4/80, newly formed vessels were visualized via CD31 staining, and nuclei were counterstained with DAPI (scale bar = 100 μm).

The early post-grafting phase represents a biologically dynamic window characterized by intense immune activity and neovascular remodeling, both of which are pivotal determinants of graft integration and long-term viability [19]. During this period, extensive loss of perilipin-positive adipocytes was confirmed from the whole-mount IF analysis at 1 week post-grafting. Notably, PKH26-labeled exosomes predominantly accumulated within infiltrating F4/80^+^ macrophages, while only limited colocalization was observed with the few remaining viable Perilipin^+^ adipocytes and CD31^+^ endothelial cells (Fig. 7).

## Discussion

Our results showed two primary constraints associated with SVF-enriched lipotransfer: a therapeutic ceiling beyond which increased cell dosages yield diminishing returns and a pronounced dose dependence that complicates clinical standardization. By directly comparing SVF supplementation with exosome-based therapy, our findings revealed that exosomes offer superior modulation of the graft microenvironment, leading to improved integration and enhanced long-term graft survival.

Effective early-phase remodeling, driven largely by immune regulation, critically determines the long-term outcomes of fat grafts [20, 21]. We observed that whole-mount IF imaging at 1 week post-grafting confirmed selective accumulation of exosomes within macrophages, indicating active participation in modulating the inflammatory microenvironment. Consistent with existing evidence, treatment with exosomes promoted macrophage polarization toward a reparative M2 phenotype, concurrently suppressing pro-inflammatory M1 macrophages implicated in tissue damage and graft degeneration [13, 22, 23]. This immunological shift was reflected by significantly reduced inflammatory infiltration observed in grafts treated with exosomes at 8-week post-grafting. The superior immunomodulatory effects of exosomes are attributable to their nanoscale dimensions and unique physicochemical properties, particularly the enhanced permeability and retention effect [24, 25], facilitating deep penetration and targeted accumulation at inflammatory sites—a capability inherently limited in SVF owing to its larger and structurally complex cellular components.

Given the pivotal role macrophages play in extracellular matrix (ECM) remodeling, early exosome-mediated immune regulation possibly has significant downstream effects on ECM organization [26, 27]. Findings from previous studies reveal that ECM organization after fat grafting is highly position-dependent [28, 29]. The presence of viable adipocytes is closely associated with dense and well-organized collagen networks, whereas adipocyte necrosis correlates with fragmented and structurally compromised ECM. Consistent with these observations, our exosome-treated grafts exhibited improved adipocyte preservation, reduced fibrosis, and decreased oil cyst formation, collectively suggesting improved ECM remodeling. These findings support our hypothesis that exosome-mediated immune modulation creates a favorable microenvironment for structured ECM remodeling, ultimately enhancing tissue quality.

An organized ECM structure is essential for providing the necessary mechanical and biochemical scaffolds that support vascular growth and adipose tissue integration [27]. However, achieving adequate neovascularization remains a major challenge for fat graft survival, as nutrient diffusion alone is insufficient to sustain central graft tissues [30–32]. SVF-enriched strategies have been proposed to enhance graft vascularization [33]. Nevertheless, their therapeutic potential is constrained by inherent biological limitations [34]. Previous studies indicate a saturable niche for transplanted stem cells, where increasing the dosage of unfractionated marrow cells fails to proportionally enhance therapeutic outcomes [35]. Furthermore, key cellular components within SVF, such as endothelial and hematopoietic cells, are highly susceptible to hypoxic stress and undergo extensive apoptosis within the initial 24 h after grafting, thereby limiting their contribution to neovascularization. While ASCs exhibit greater hypoxia tolerance, their viability is largely confined to within 300 microns of the graft surface, where oxygen and nutrients can diffuse from surrounding tissues, restricting their capacity to support vascularization in deeper graft regions [32].

In contrast, exosomes present a promising non-cellular alternative that can overcome these limitations. Their nanoscale size facilitates uniform intragraft distribution through microcapillary networks, enabling effective delivery to hypoxic central regions inaccessible to transplanted cells. Additionally, encapsulation within exosomes protects bioactive molecules from rapid degradation, enabling sustained biological function *in vivo* [36]. Beyond these biological advantages, exosomes offer practical benefits that support clinical translation. They can be produced in large quantities through serial expansion of donor cells, stored at − 80 °C with minimal loss of function [37], and administered intravenously to achieve systemic distribution, including bypassing pulmonary filtration and penetrating the blood-brain barrier [38]. Furthermore, exosomes mitigate key safety concerns associated with cell-based therapies, including immune rejection, maldifferentiation, and tumor formation. Collectively, our findings provide compelling evidence supporting the translational development of exosome-based strategies for clinical fat grafting applications.

A primary limitation of this study was the challenge in establishing truly comparable concentrations between exosomes and SVF cells, considering that exosomes are measured using protein volume while SVF cells are quantified using cell count. Recognizing that an equivalent concentration is practically unattainable, we conducted *in vitro* experiments to identify an effective exosome concentration for comparison with the optimal SVF concentration. In addition, semi-quantitative fluorescence intensity analysis revealed a notable reduction in exosome abundance after 4-week post-grafting, which showed a finite exosome lifespan in local fat grafts. Therefore, optimizing dosing strategies for exosome administration is required in further investigation. Moreover, the sample size and observation period were limited in our study. A long-term evaluation of the two fat graft methods would provide more valuable insights for the clinical translation of exosome-based therapies. Nevertheless, the use of three-dimensional reconstruction following micro-CT imaging provided a precise method for quantifying graft volume, representing a methodological strength particularly suited to small-volume fat grafting models.

## Conclusions

Compared with those of SVF cells, exosomes showed superior effects in improving fat graft retention, reducing inflammatory infiltration, preserving the functional adipose structure, and promoting angiogenesis in the local grafted tissue, suggesting that fat co-transplanted with exosomes is a potential alternative strategy for SVF-enriched lipotransfer.

## Supplementary Information

Below is the link to the electronic supplementary material.

Supplementary file1 (TIF 876 KB)

Supplementary file2 (TIF 16463 KB)

Supplementary file3 (TIF 3944 KB)

Supplementary file4 (TIF 22314 KB)

Supplementary file5 (TIF 5975 KB)

## References

[1] Zhou Y, Wang J, Li H, et al. Efficacy and safety of cell-assisted lipotransfer: a systematic review and meta-analysis. Plast Reconstr Surg. 2016;137:44e. 10.1097/PRS.0000000000001981. 26710060 10.1097/PRS.0000000000001981

[2] Planat-Benard V, Silvestre J-S, Cousin B, et al. Plasticity of human adipose lineage cells toward endothelial cells: physiological and therapeutic perspectives. Circulation. 2004;109:656–63. 10.1161/01.CIR.0000114522.38265.61. 14734516 10.1161/01.CIR.0000114522.38265.61

[3] Hong KY, Yim S, Kim HJ, et al. The fate of the adipose-derived stromal cells during angiogenesis and adipogenesis after cell-assisted lipotransfer. Plast Reconstr Surg. 2018;141:365. 10.1097/PRS.0000000000004021. 29036025 10.1097/PRS.0000000000004021

[4] Kapur SK, Katz AJ. Review of the adipose derived stem cell secretome. Biochimie. 2013;95:2222–8. 10.1016/j.biochi.2013.06.001. 23770442 10.1016/j.biochi.2013.06.001

[5] Rehman J, Traktuev D, Li J, et al. Secretion of angiogenic and antiapoptotic factors by human adipose stromal cells. Circulation. 2004;109:1292–8. 10.1161/01.CIR.0000121425.42966.F1. 14993122 10.1161/01.CIR.0000121425.42966.F1

[6] Hong KY, Kim I-K, Park SO, et al. Systemic administration of adipose-derived stromal cells concurrent with fat grafting. Plast Reconstr Surg. 2019;143:973e–82e. 10.1097/PRS.0000000000005513. 30807495 10.1097/PRS.0000000000005513

[7] Berkowitz AL, Miller MB, Mir SA, et al. Glioproliferative lesion of the spinal cord as a complication of “stem-cell tourism.” N Engl J Med. 2016;375:196–8. 10.1056/NEJMc1600188. 27331440 10.1056/NEJMc1600188

[8] Aguilar S, Nye E, Chan J, et al. Murine but not human mesenchymal stem cells generate osteosarcoma-like lesions in the lung. Stem Cells. 2007;25:1586–94. 10.1634/stemcells.2006-0762. 17363552 10.1634/stemcells.2006-0762

[9] Haarer J, Johnson CL, Soeder Y, Dahlke MH. Caveats of mesenchymal stem cell therapy in solid organ transplantation. Transplant Int. 2015;28:1–9. 10.1111/tri.12415. 10.1111/tri.12415 25082213

[10] Paik KJ, Zielins ER, Atashroo DA, et al. Studies in fat grafting: Part V. Cell-assisted lipotransfer to enhance fat graft retention is dose dependent. Plast Reconstr Surg. 2015;136:67–75. 10.1097/PRS.0000000000001367. 25829158 10.1097/PRS.0000000000001367 PMC4483157

[11] Poulos J. The limited application of stem cells in medicine: a review. Stem Cell Res Ther. 2018;9:1. 10.1186/s13287-017-0735-7. 29291747 10.1186/s13287-017-0735-7 PMC5749007

[12] Chen B, Cai J, Wei Y, et al. Exosomes are comparable to source adipose stem cells in fat graft retention with up-regulating early inflammation and angiogenesis. Plast Reconstr Surg. 2019;144:816e–27e. 10.1097/PRS.0000000000006175. 31385891 10.1097/PRS.0000000000006175

[13] Wang Z, Chen Y, Zhu S, et al. The effects of macrophage-mediated inflammatory response to the donor site on long-term retention of a fat graft in the recipient site in a mice model. J Cell Physiol. 2020;235:10012–23. 10.1002/jcp.29816. 32557574 10.1002/jcp.29816

[14] Chiu C-H. Does stromal vascular fraction ensure a higher survival in autologous fat grafting for breast augmentation? A volumetric study using 3-dimensional laser scanning. Aesthet Surg J. 2019;39:41–52. 10.1093/asj/sjy030. 29438465 10.1093/asj/sjy030

[15] Roshdy OH, Abdallah WI, Farid CI, et al. Stromal vascular fraction improves the durability of autologous fat temple augmentation-a split-face randomized study using ultrasound biomicroscopy. J Plast Reconstr Aesthet Surg. 2022;75:1870–7. 10.1016/j.bjps.2021.12.005. 35125305 10.1016/j.bjps.2021.12.005

[16] Jeon HJ, Choi DH, Lee JH, et al. A prospective study of the efficacy of cell-assisted lipotransfer with stromal vascular fraction to correct contour deformities of the autologous reconstructed breast. Aesthet Plast Surg. 2021;45:853–63. 10.1007/s00266-020-01981-y. 10.1007/s00266-020-01981-y 32995982

[17] Zuk PA, Zhu M, Mizuno H, et al. Multilineage cells from human adipose tissue: implications for cell-based therapies. Tissue Eng. 2001;7:211–28. 10.1089/107632701300062859. 11304456 10.1089/107632701300062859

[18] Atik B, Öztürk G, Erdogan E, Tan Ö. Comparison of techniques for long-term storage of fat grafts: an experimental study. Plast Reconstr Surg. 2006;118:1533. 10.1097/01.prs.0000240806.19404.a8. 17102724 10.1097/01.prs.0000240806.19404.a8

[19] Kato H, Mineda K, Eto H, et al. Degeneration, regeneration, and cicatrization after fat grafting: dynamic total tissue remodeling during the first 3 months. Plast Reconstr Surg. 2014;133:303e. 10.1097/PRS.0000000000000066. 24572875 10.1097/PRS.0000000000000066

[20] Chen X, Chen W, Xu H, et al. Disulfiram improves fat graft retention by modulating macrophage polarization with inhibition of NLRP3 inflammasome-mediated pyroptosis. Aesthet Surg J. 2024;44:NP501–18. 10.1093/asj/sjae075. 38567442 10.1093/asj/sjae075 PMC11177556

[21] Li Y, Chen X, Liu L, et al. Alternatively activated macrophages at the recipient site improve fat graft retention by promoting angiogenesis and adipogenesis. J Cell Mol Med. 2022;26:3235–42. 10.1111/jcmm.17330. 35570832 10.1111/jcmm.17330 PMC9170812

[22] Hao X, Guo Y, Wang R, et al. Exosomes from adipose-derived mesenchymal stem cells promote survival of fat grafts by regulating macrophage polarization via let-7c. Acta Biochim Biophys Sin (Shanghai). 2021;53:501–10. 10.1093/abbs/gmab018. 33704368 10.1093/abbs/gmab018

[23] Zhu Y, Zhang J, Hu X, et al. Supplementation with extracellular vesicles derived from adipose-derived stem cells increases fat graft survival and browning in mice: a cell-free approach to construct beige fat from white fat grafting. Plast Reconstr Surg. 2020;145:1183. 10.1097/PRS.0000000000006740. 32332538 10.1097/PRS.0000000000006740

[24] Lee W-H, Loo C-Y, Traini D, Young PM. Nano- and micro-based inhaled drug delivery systems for targeting alveolar macrophages. Expert Opin Drug Deliv. 2015;12:1009–26. 10.1517/17425247.2015.1039509. 25912721 10.1517/17425247.2015.1039509

[25] Kooijmans SAA, Vader P, van Dommelen SM, et al. Exosome mimetics: a novel class of drug delivery systems. Int J Nanomedicine. 2012;7:1525–41. 10.2147/IJN.S29661. 22619510 10.2147/IJN.S29661 PMC3356169

[26] Wang L, Hu L, Zhou X, et al. Exosomes secreted by human adipose mesenchymal stem cells promote scarless cutaneous repair by regulating extracellular matrix remodelling. Sci Rep. 2017;7:13321. 10.1038/s41598-017-12919-x. 29042658 10.1038/s41598-017-12919-x PMC5645460

[27] Crewe C, An YA, Scherer PE. The ominous triad of adipose tissue dysfunction: inflammation, fibrosis, and impaired angiogenesis. J Clin Invest. 2017;127:74–82. 10.1172/JCI88883. 28045400 10.1172/JCI88883 PMC5199684

[28] Cai J, Li B, Liu K, et al. Macrophage infiltration regulates the adipose ECM reconstruction and the fibrosis process after fat grafting. Biochem Biophys Res Commun. 2017;490:560–6. 10.1016/j.bbrc.2017.06.078. 28625922 10.1016/j.bbrc.2017.06.078

[29] Chen X, Deng Z, Feng J, et al. Necroptosis in macrophage foam cells promotes fat graft fibrosis in mice. Front Cell Dev Biol. 2021. 10.3389/fcell.2021.651360. 33842478 10.3389/fcell.2021.651360 PMC8027326

[30] Yoshimura K, Eto H, Kato H, et al. In vivo manipulation of stem cells for adipose tissue repair/reconstruction. Regen Med. 2011;6:33–41. 10.2217/rme.11.62. 21999260 10.2217/rme.11.62

[31] Suga H, Eto H, Aoi N, et al. Adipose tissue remodeling under ischemia: death of adipocytes and activation of stem/progenitor cells. Plast Reconstr Surg. 2010;126:1911. 10.1097/PRS.0b013e3181f4468b. 21124131 10.1097/PRS.0b013e3181f4468b

[32] Eto H, Kato H, Suga H, et al. The fate of adipocytes after nonvascularized fat grafting: evidence of early death and replacement of adipocytes. Plast Reconstr Surg. 2012;129:1081. 10.1097/PRS.0b013e31824a2b19. 22261562 10.1097/PRS.0b013e31824a2b19

[33] Zakhari JS, Zabonick J, Gettler B, Williams SK. Vasculogenic and angiogenic potential of adipose stromal vascular fraction cell populations in vitro. In Vitro Cell Dev Biol Anim. 2017;54:32. 10.1007/s11626-017-0213-7. 29197029 10.1007/s11626-017-0213-7 PMC5760587

[34] Tissiani L, Alonso N. A prospective and controlled clinical trial on stromal vascular fraction enriched fat grafts in secondary breast reconstruction. Stem Cells Int. 2016;2016:2636454. 10.1155/2016/2636454. 26962306 10.1155/2016/2636454 PMC4707337

[35] Marino R, Martinez C, Boyd K, et al. Transplantable marrow osteoprogenitors engraft in discrete saturable sites in the marrow microenvironment. Exp Hematol. 2008;36:360–8. 10.1016/j.exphem.2007.11.002. 18179857 10.1016/j.exphem.2007.11.002 PMC3391997

[36] Zeng H, Guo S, Ren X, et al. Current strategies for exosome cargo loading and targeting delivery. Cells. 2023;12:1416. 10.3390/cells12101416. 37408250 10.3390/cells12101416 PMC10216928

[37] Ahmadian S, Jafari N, Tamadon A, et al. Different storage and freezing protocols for extracellular vesicles: a systematic review. Stem Cell Res Ther. 2024;15:453. 10.1186/s13287-024-04005-7. 39593194 10.1186/s13287-024-04005-7 PMC11600612

[38] Zhang Z-W, Wei P, Zhang G-J, et al. Intravenous infusion of the exosomes derived from human umbilical cord mesenchymal stem cells enhance neurological recovery after traumatic brain injury via suppressing the NF-κB pathway. Open Life Sci. 2022;17:189–201. 10.1515/biol-2022-0022. 35415238 10.1515/biol-2022-0022 PMC8932398

