# Supplementary figures and images for: Exosome-Mediated Enhancement of Fat Graft Retention: A Comparative Preclinical Study with Stromal Vascular Fraction
Source: Aesthetic Plast Surg. 2025 Nov 17;49(24):6912–22. doi: 10.1007/s00266-025-05350-5 (PMC12855394; doi:10.1007/s00266-025-05350-5)

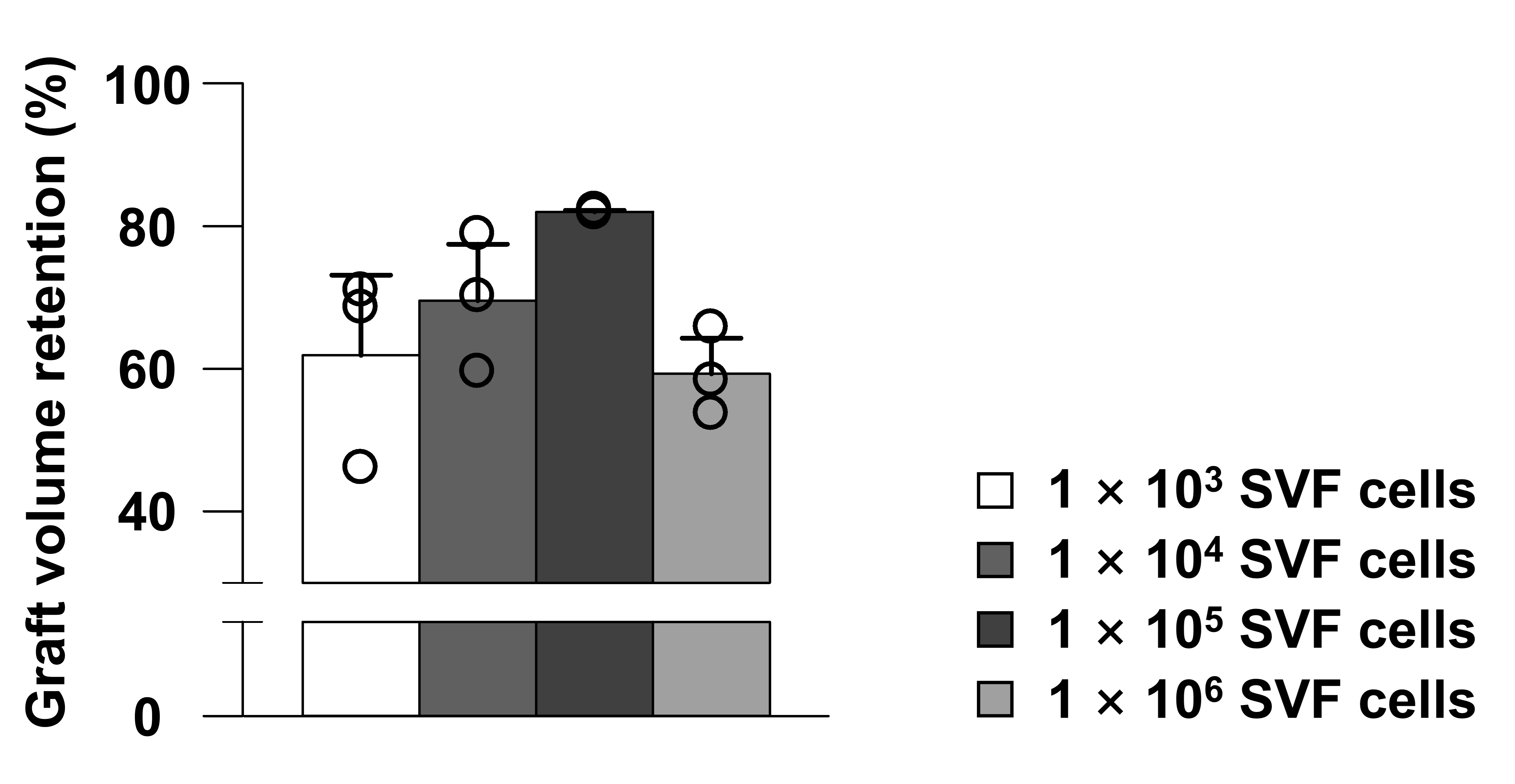

Supplement: Supplementary file 1 — Supplementary file1 (TIF 876 KB) [file 266_2025_5350_MOESM1_ESM.tif]

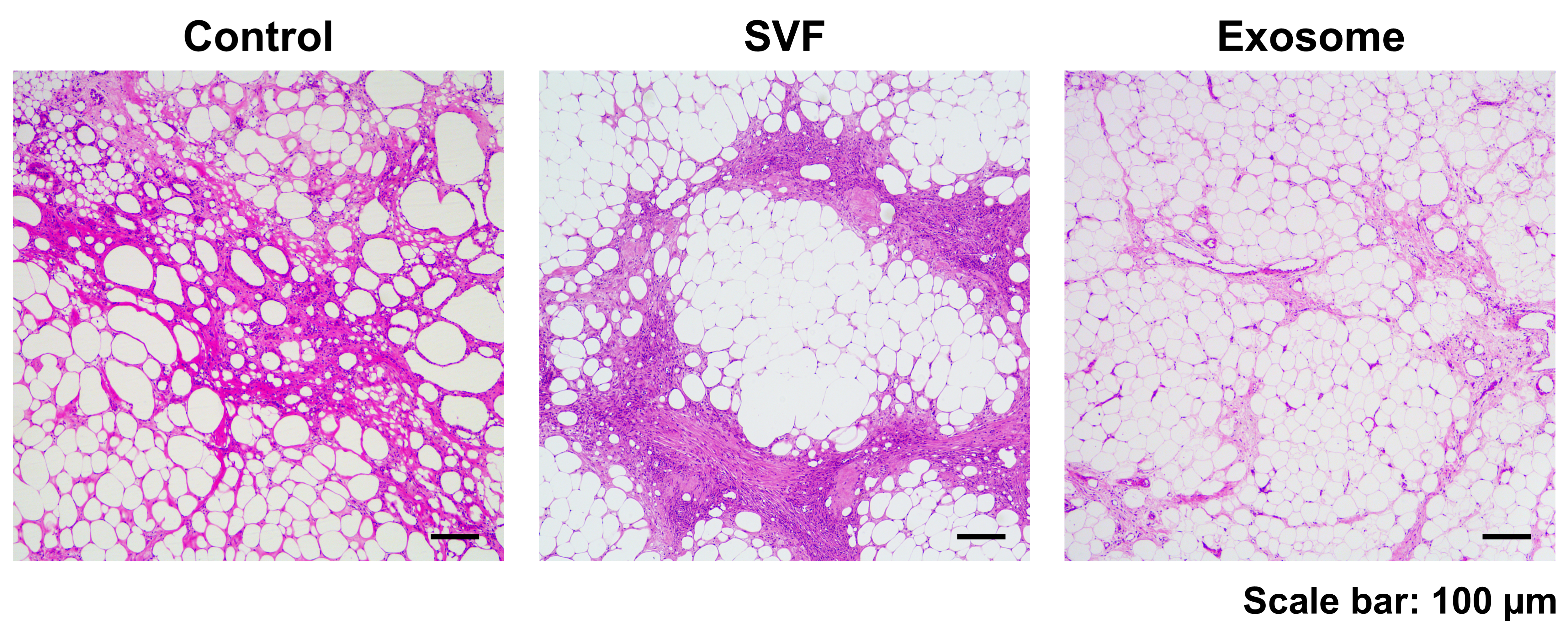

Supplement: Supplementary file 2 — Supplementary file2 (TIF 16463 KB) [file 266_2025_5350_MOESM2_ESM.tif]

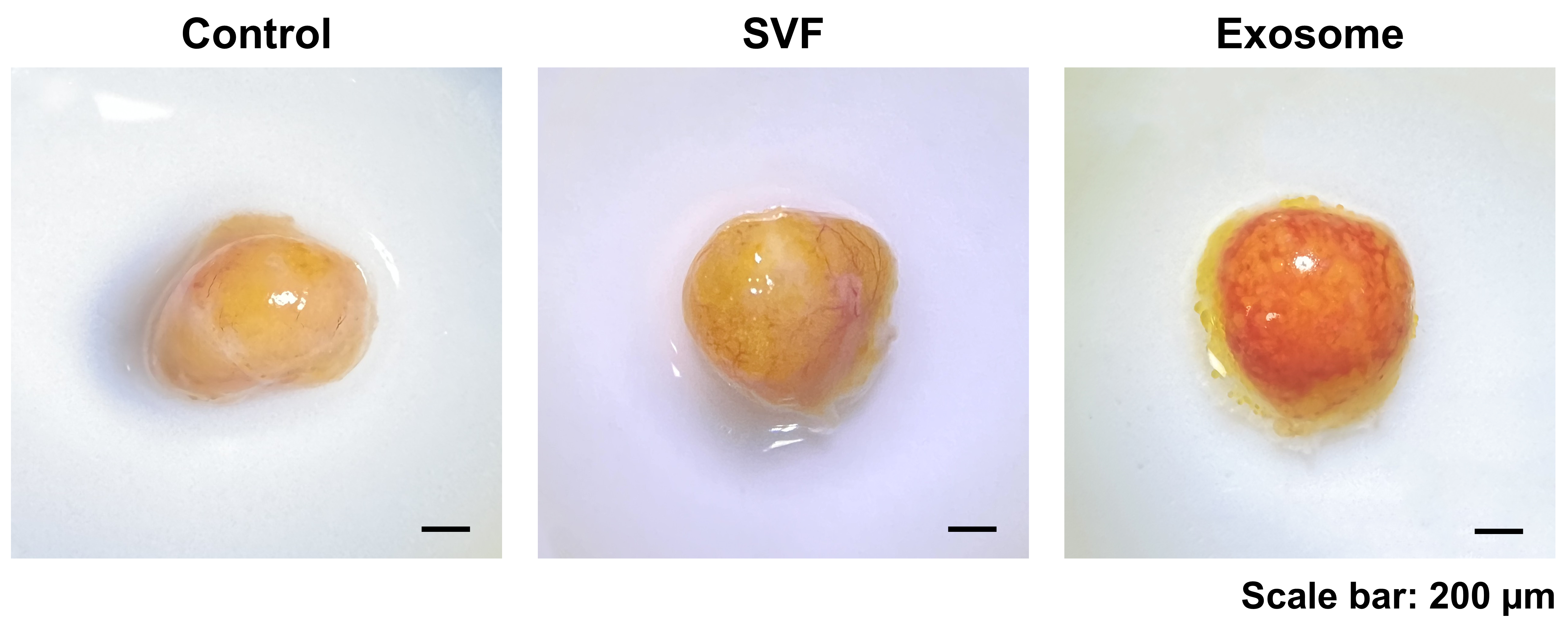

Supplement: Supplementary file 3 — Supplementary file3 (TIF 3944 KB) [file 266_2025_5350_MOESM3_ESM.tif]

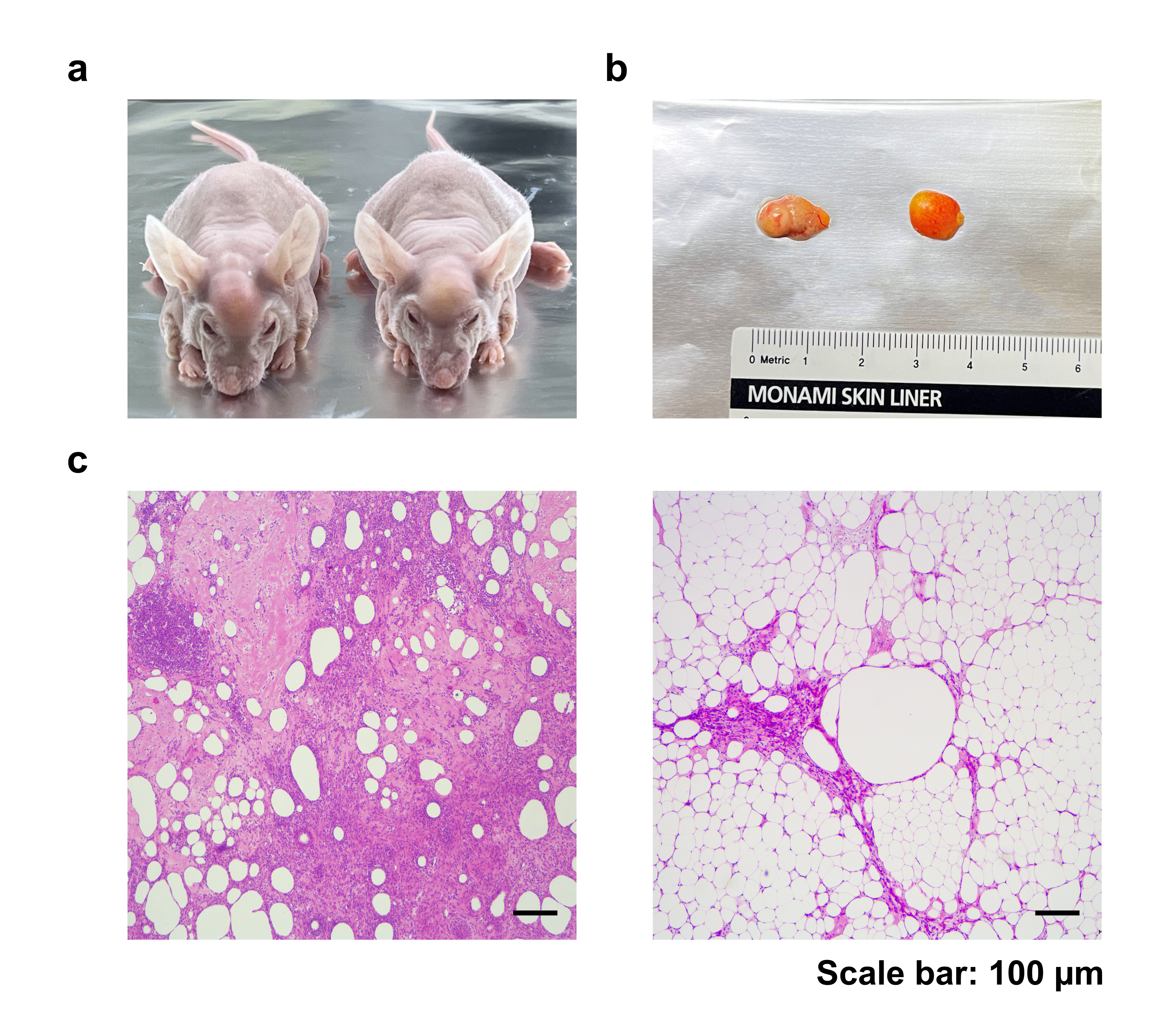

Supplement: Supplementary file 4 — Supplementary file4 (TIF 22314 KB) [file 266_2025_5350_MOESM4_ESM.tif]

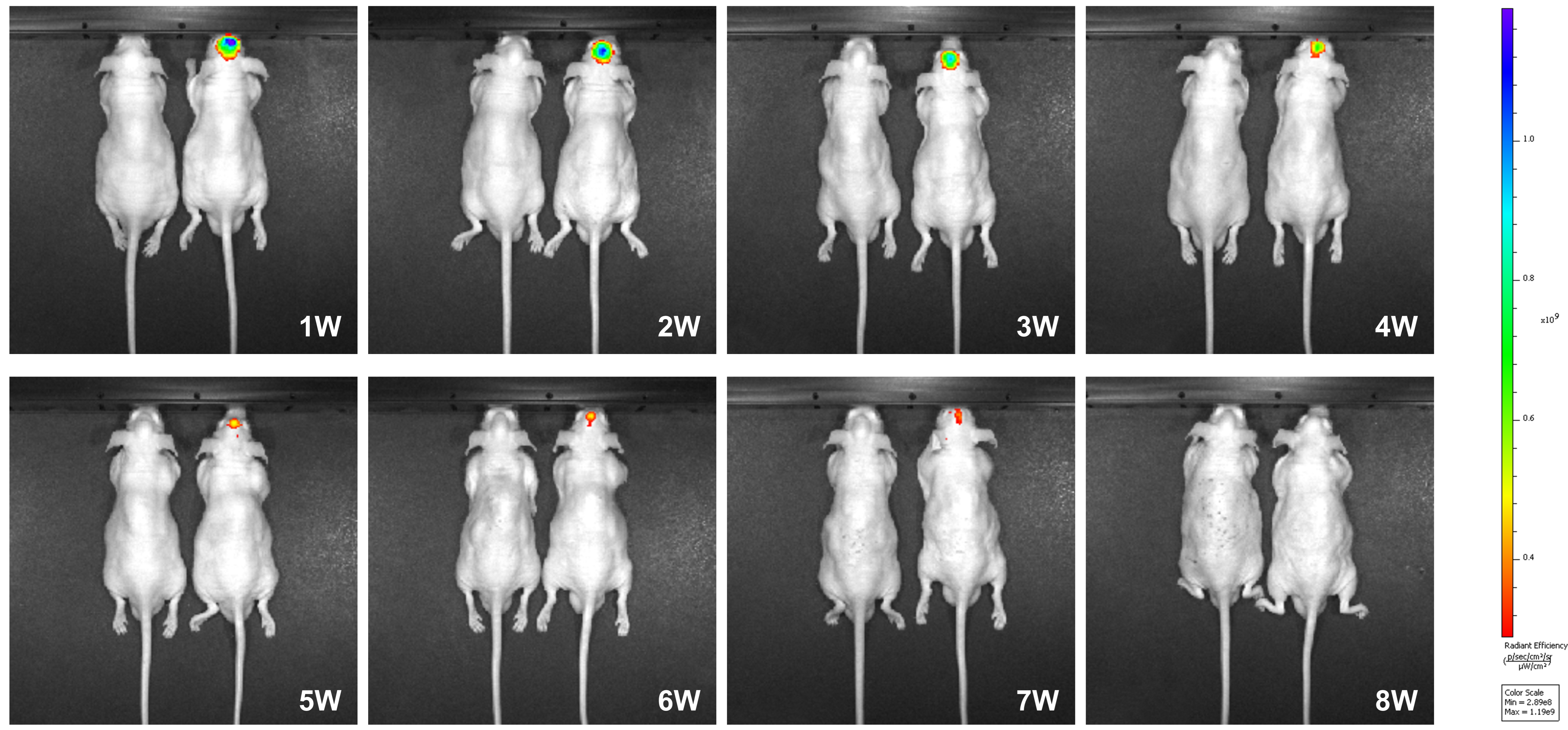

Supplement: Supplementary file 5 — Supplementary file5 (TIF 5975 KB) [file 266_2025_5350_MOESM5_ESM.tif]
